# Identification of a Novel Pathogenic ABCC6 Mutation Through Familial Genetic Analysis in Pseudoxanthoma Elasticum: A Case Report

**DOI:** 10.7759/cureus.101655

**Published:** 2026-01-16

**Authors:** Likeng Liang, Runduo Li, Dan Ren, Yong Zhou

**Affiliations:** 1 School of Medicine, Nankai University, Tianjin, CHN; 2 Department of Dermatology, The First Medical Center, People's Liberation Army (PLA) General Hospital, Beijing, CHN; 3 Department of Dermatology, Chinese People's Liberation Army (PLA) Medical School, Beijing, CHN

**Keywords:** abcc6 protein, genetic testing, mutation, prenatal counseling, pseudoxanthoma elasticum, variant

## Abstract

This study conducted genetic analysis on a patient clinically diagnosed with pseudoxanthoma elasticum (PXE) and their family to identify pathogenic mutations in the ABCC6 gene and analyze its inheritance pattern. We performed whole-exome sequencing of the ABCC6 gene on peripheral blood samples from the proband, his parents, and his sister, with validation via Sanger sequencing. Results revealed two heterozygous mutations in the proband: a previously reported missense mutation c.3412C>T (p. Arg1138Trp) and an unreported frameshift deletion mutation c.3160_3161del (p. Thr1054Glyfs*2). Both variants were classified as pathogenic according to the American College of Medical Genetics and Genomics (ACMG) guidelines. The c.3160_3161del mutation represents a novel pathogenic variant. The proband's mother carried the c.3412C>T mutation, while the father and sister carried the c.3160_3161del mutation. Among four family members, only the proband has been diagnosed with elastic fibrillary pseudoxanthoma. This pattern aligns with the typical autosomal recessive inheritance of PXE, with the proband being compound heterozygous. The findings of this study not only expand the spectrum of pathogenic mutations in the ABCC6 gene but also provide clear molecular evidence for genetic counselling and prenatal diagnosis.

## Introduction

Pseudoxanthoma elasticum (PXE), also known as Gronblad-Strandberg syndrome, is a rare autosomal recessive connective tissue disorder [1] mainly caused by pathogenic variants in the ABCC6 gene, leading to calcification and fragmentation of elastic fibers in multiple systems such as the skin, retina, and vasculature. It is a multisystem disease [2]. The estimated clinical prevalence of PXE ranges from approximately 1 in 100,000 to 1 in 25,000 individuals, with a noted female predominance [3]. Although PXE is a congenital disorder, its clinical manifestations typically become apparent from childhood to early adulthood [4]. In most cases, cutaneous changes are the initial sign of PXE [5]. Cutaneous manifestations of PXE most commonly occur in the neck and underarm area, characterized initially by thickened skin and yellowish papules, and progressing to loose, sagging folds in advanced stages. The skin rash can also occur at atypical locations, such as on the skin of the upper or lower limbs away from flexures or in the perineal area. Characteristic changes of PXE rarely appear on the face. Adult patients may develop suggestive diagonal chin wrinkles, but this has never been pathologically confirmed [6]. Ocular involvement typically manifests as peripapillary vascular striations. Cardiovascular compromise arises mainly from arterial degeneration and calcification [7].

The primary histopathological hallmark of PXE is the degeneration of elastic fibers, which undergo progressive mineralization and fragmentation, resulting in a histological pattern of disrupted elastic fibers. In the skin of PXE patients, the elastic fibers in the mid-dermis are typically polymorphic, mineralized, and fragmented. Histopathological examination of a skin biopsy remains the gold standard for the clinical diagnosis of PXE. The primary histopathological hallmark of PXE is the degeneration of elastic fibers, which undergo progressive mineralization and fragmentation, resulting in a histological pattern of disrupted elastic fibers. The elastic fibers in the mid-dermis are typically polymorphic, mineralized, and fragmented. Histopathological examination of a skin biopsy remains the gold standard for the clinical diagnosis of PXE [8].

The diagnosis of PXE is established through a set of multi-system criteria. A definitive diagnosis requires the presence of two or more major criteria from different categories (skin, eye, vascular, genetics, or biology). Major criteria include specific findings such as characteristic yellowish neck papules/plaques with biopsy-confirmed calcified elastic fibers, bilateral *peau d'orange* retina with definitive angioid streaks, early-onset arterial calcification or peripheral artery disease, biallelic pathogenic variants in relevant genes (ABCC6, CYP2U1, ENPP1, GGCX), or a severe reduction (≥70%) in plasma pyrophosphate (PPi). A probable diagnosis, warranting further investigation, is made with one major criterion plus one or more minor criteria. Minor criteria encompass less specific signs like atypical retinal angioid streaks, isolated organ calcifications (e.g., spleen, liver), or a moderate reduction (40%-69%) in plasma PPi [5].

Numerous pieces of evidence indicate that the pathogenesis of PXE is strongly associated with mutations in the ABCC6 gene [9]. In the majority of patients with PXE, loss-of-function mutations in ABCC6 can be detected by advanced sequencing technologies. To date, more than 300 pathogenic variants in ABCC6 have been documented worldwide. The majority of these variants are single-nucleotide variants, predominantly missense variants [10]. In this study, we investigated a case of PXE and the patient's family members. We identified a novel pathogenic mutation in the ABCC6 gene through genetic sequencing.

## Case presentation

**Figure 1:**
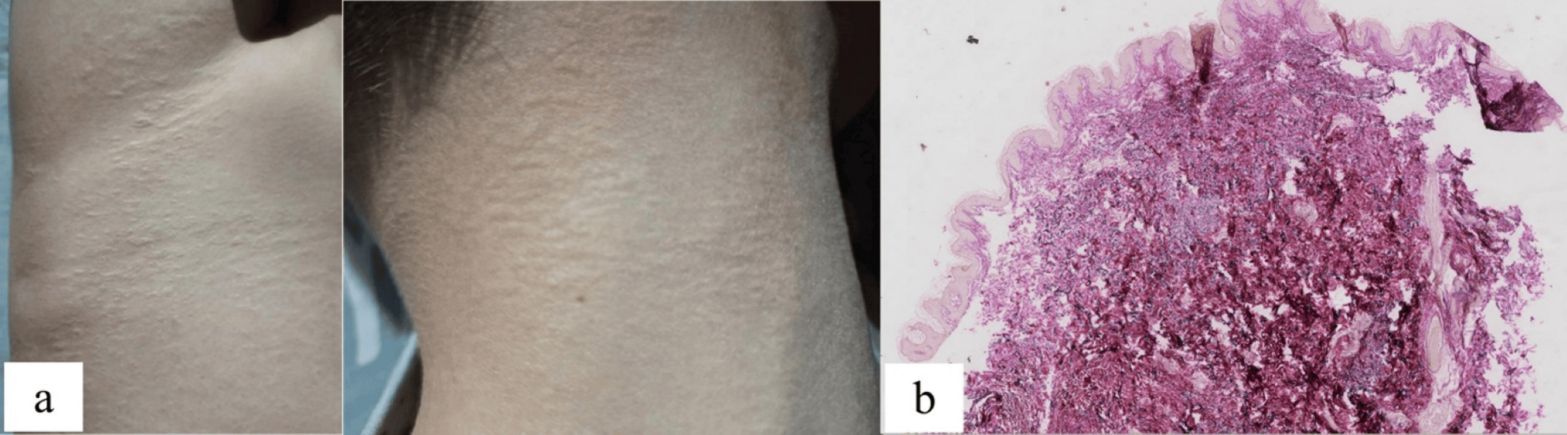
Clinical manifestations of the proband. (a) Yellowish papules in the armpits and neck. (b) The pathology showed that the elastic fibers in the dermis were thickened, broken, and disordered (elastic fibers staining × 50).

The patient was a 12-year-old male who presented with yellowish papules on the abdomen, groin, and neck. The lesions initially appeared on the abdomen and bilateral groin 10 years ago without an obvious cause. The papules followed skin cleavage lines and were not associated with pain, pruritus, or other subjective symptoms. Similar rashes emerged on the neck four years later. During the course of the disease, the skin lesions progressed slowly but never resolved spontaneously. Physical examination showed that yellowish papules were distributed along the skin lines on the neck, lower abdomen, and groin. The papules ranged in size from 1 to 3 mm, were soft in consistency, and had well-defined borders (Figure 1a). Physical exam was unremarkable, including a full range of motion in major joints. Abdominal ultrasound and cardiovascular assessment were normal. Dermoscopy revealed reddish-brown patches with a homogeneous yellow structure and marginal telangiectasia on the neck. Fundus examination revealed angioid streaks in the fundus. Histopathological examination showed that elastic fibers in the dermis were thickened, broken, and disordered (Figure 1b). Laboratory findings were unremarkable. There was no parental consanguinity. Neither his parents nor his sister exhibited similar clinical manifestations. The patient had no personal history of hypertension, diabetes, or infectious diseases, and no family history of similar disorders.

Subjects

The study cohort comprised a 12-year-old male proband diagnosed with PXE at the Chinese PLA General Hospital, along with his biological parents and younger sister. All participants provided verbal agreement and signed medical consent for the diagnostic procedures, including whole-genome sequencing. For minor participants, parental or guardian consent was obtained for all related examinations to ensure complete ethical adherence.

DNA extraction

We collected 2 mL of venous blood from each subject into ethylenediaminetetraacetic acid (EDTA)-anticoagulated tubes for further processing. Genomic DNA was extracted from the blood samples using the Qiagen FlexiGene DNA Kit (Cat. No. 51206; Qiagen, Germany). DNA quantification was performed using a Nanodrop 2000 Ultra-Micro Spectrophotometer (Thermo Fisher Scientific, Waltham, MA) to ensure that the OD 260/280 ratios were within the range of 1.8-2.0, DNA concentrations ranged from 100 to 500 ng/μL, and the total genomic DNA yield exceeded 3.5 μg. Genomic integrity was simultaneously assessed by agarose gel electrophoresis.

Probe design

Referring to the OMIM and HGMD databases, we designed 31 capture probes targeting the exons of ABCC6 using Agilent SureDesign (Agilent, Santa Clara, CA) through the online tool.

Construction and amplification of genomic DNA libraries

The SureSelect Target Enrichment System Target Sequence Capture Kit (Agilent, Santa Clara, CA) was used to construct target gene capture libraries from the genomic DNA samples. Amplification was performed under the following conditions: initial denaturation at 95 °C for 10 minutes (1 cycle); 35 cycles of denaturation at 95 °C for 30 seconds, annealing at 60 °C for 30 seconds, and extension at 72 °C for 45 seconds; followed by a final extension at 72 °C for 5 minutes (1 cycle). The resulting DNA library was purified using AMPure XP magnetic beads. Library quality and quantity were assessed using the apparatus 2100 Bioanalyzer with the DNA 1000 Kit.

Hybridization and capture

The prepared genomic DNA library was hybridized with target-specific capture probes. Following hybridization, streptavidin-coated magnetic beads were employed to isolate the bound target DNA fragments.

Sequencing

Gene sequencing was performed on the NovaSeq platform (Illumina, San Diego, CA) in strict accordance with the manufacturer’s instructions.

Data analysis

Sequencing data were converted from BCL to FASTQ format using bcl2fastq (v2.0). Reads were aligned to the human reference genome (GRCh37/hg19) using BWA (v0.7.15). Quality control was performed based on global alignment depth, regional alignment depth, regional coverage, and locus-specific alignment. Single-nucleotide variants (SNVs) and small insertions and deletions (InDels) were detected using GATK (v3.6). Putative copy number variations (CNVs) were analyzed using CODEX, XHMM (v1.0), and KSCNV. Detected variants were subsequently annotated and classified according to the American College of Medical Genetics and Genomics (ACMG) guidelines into the following five categories: pathogenic, likely pathogenic, uncertain significance, likely benign, and benign. Pathogenicity evidence included PVS1, PS1-PS4, PM1-PM6, and PP1-PP5; evidence supporting benign classification included BA1, BS1-BS4, and BP1-BP6 [11-13].

Sanger sequencing validation

We employed Primer Z software to design primers for the detected single genes. Following synthesis and dilution, PCR amplification was performed under optimized conditions using the Goldstar Taq mix on the ABI PCR amplification instrument. The PCR products were then subjected to 1% agarose gel electrophoresis for identification, purification, and recycling of the products. The recovered products were then subjected to primary sequencing (ABI 3730 Primary Sequencer). The primary sequencing results were analyzed, and false-positive variants were excluded through bidirectional sequencing verification (Table 1).

**Table 1:** Primer sequences.

Gene	Primer sequences (5′-3′)	
	Forward	Reverse
c.3412C>T	AGTCGCGGGAAACTGATCCT	GGGCTCTCTGTGCTTCTGGA
c.3160_3161del	CCTGACCTCTCCGTACCTGA	CCAGGTTGCTCTTCCAGAGG

Mutation analysis

**Figure 2:**
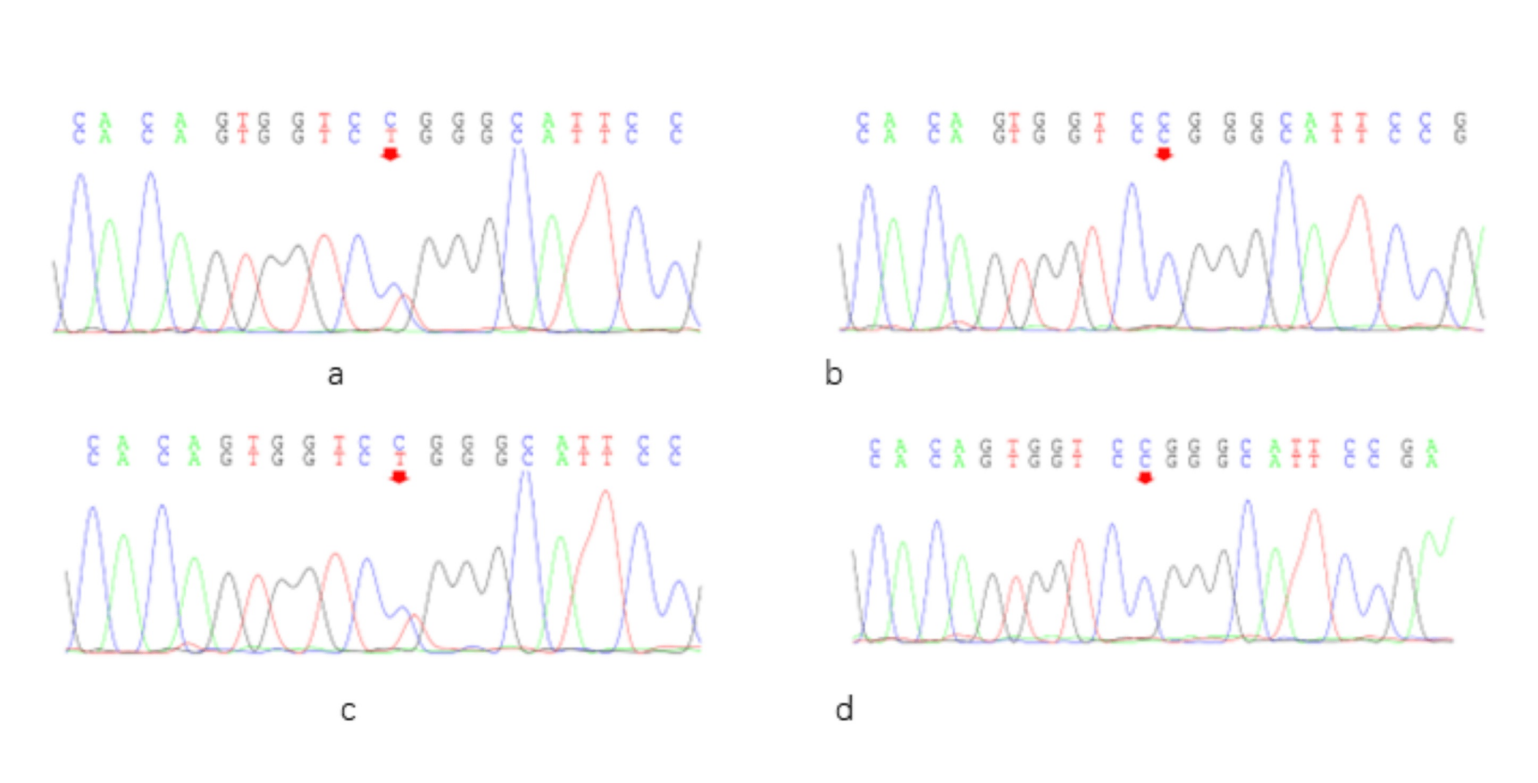
Family screening for the c.3412C>T gene mutation. The c.3412C>T heterozygote mutation was present in the proband (a) and his mother (c), but was not found in his father (b) and sister (d).

**Figure 3:**
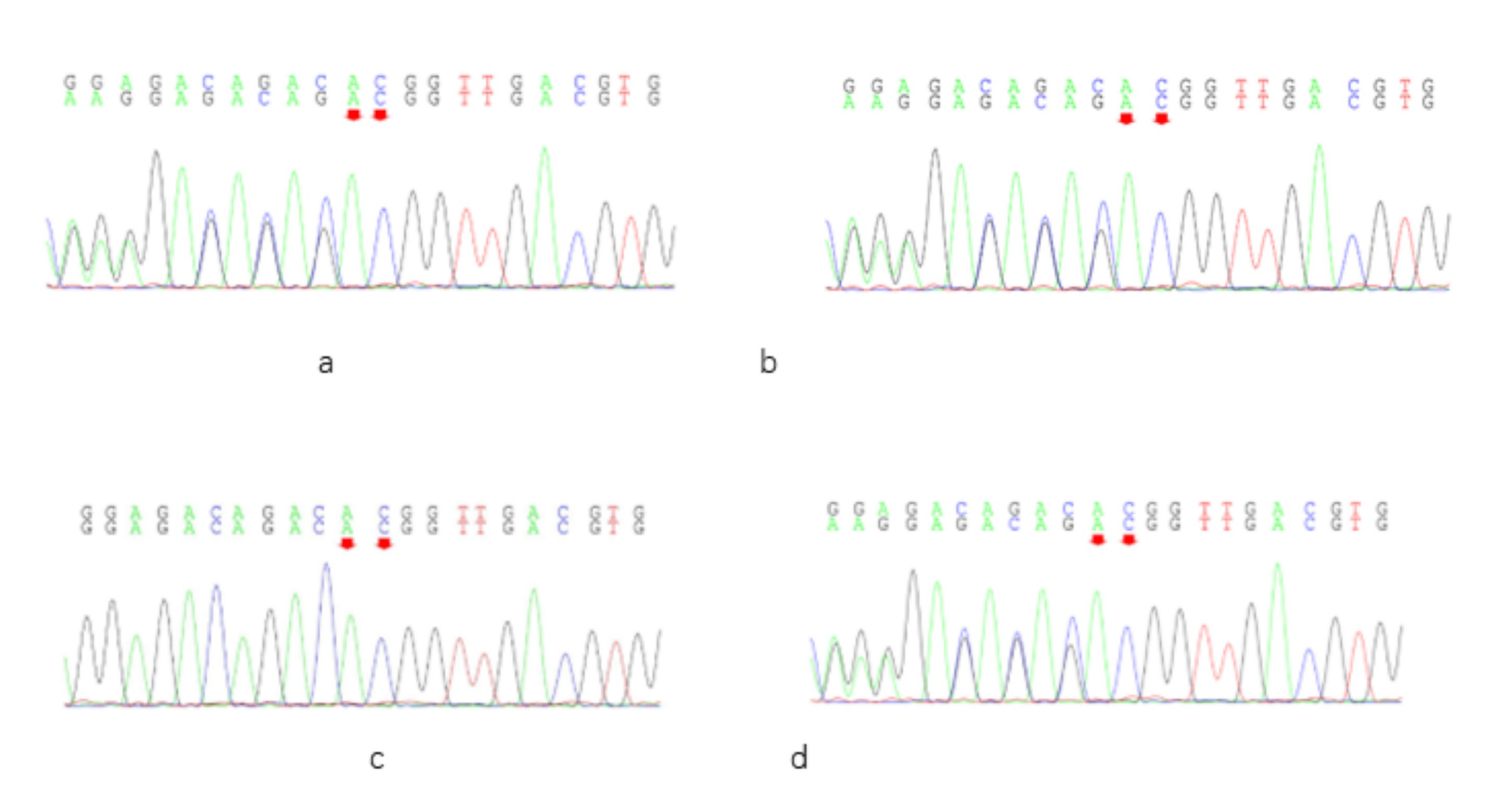
Family screening for the c.3160_3161del gene mutation. The c.3160_3161del heterozygous mutation was present in the proband (a), his father (b), and his sister (d), but was not found in his mother (c).

**Figure 4:**
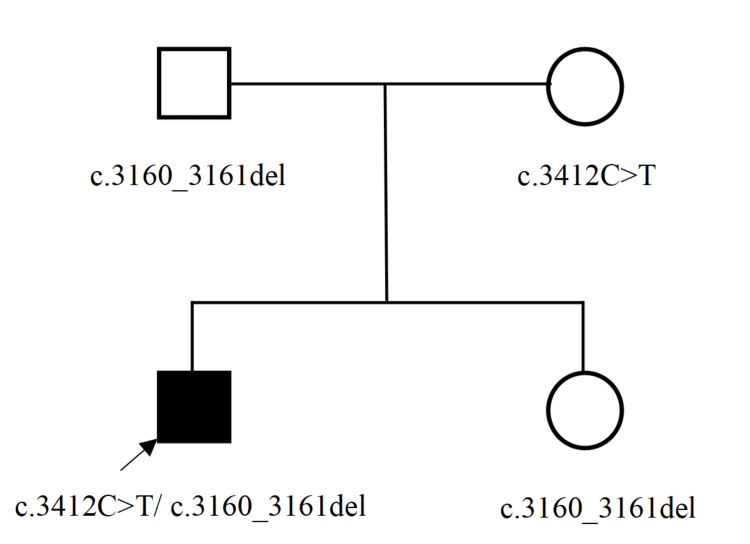
Lineage map of ABCC6 gene mutations in the proband and his family. Testing identified the proband’s father and sister as carriers of the ABCC6 c.3160_3161del variant, and his mother as a carrier of the c.3412C>T variant. The proband was compound heterozygous for both variants. Clinically, he was the only affected individual in the family.

A total of two ABCC6 gene mutations, c.3412C>T and c.3160_3161del, were identified in the subjects by next-generation sequencing (NGS). The c.3412C>T heterozygous mutation, previously reported in the literature [14], was detected in both the proband and his mother (Figure 2). This mutation is located in exon 24 of the ABCC6 gene and results in a missense variant (p. Arg1138Trp), which is predicted to be pathogenic according to the ACMG Variant Classification Guidelines (supporting evidence: PM3_VeryStrong, PM1, PM2, PP3). The c.3160_3161del heterozygous mutation was identified in the proband, his father, and his sister (Figure 3). This variant occurs at chromosomal position chr16:16259625 and causes a frameshift mutation (p. Thr1054Glyfs*2), which is also classified as pathogenic based on the ACMG guidelines (supporting evidence: PVS1_VeryStrong, PM2, PM3). Genetic analysis of this family’s pedigree revealed that PXE is inherited in an autosomal recessive pattern in this family, with the identified causative variants in a heterozygous state (Figure 4).

## Discussion

PXE is a rare genetic disorder that typically presents with characteristic lesions in the skin, ocular fundus, and vascular system. The diagnosis is established based on a combination of clinical features from dermatological and ophthalmological examinations, supported by histopathological findings and molecular genetic analysis [15]. The proband in this study is a 12-year-old male who exhibited classic cutaneous manifestations of PXE, satisfying the major diagnostic criteria.

Current researches have confirmed that the pathogenesis of PXE is associated with ABCC6 gene mutations, which are located on the short arm of chromosome 16 (16p13.1) and consist of 31 exons encoding a transmembrane transporter protein of 1503 amino acids (ABCC6 protein) [16]. ABCC6 is primarily expressed on the basolateral membrane of hepatic and renal cells and contains three transmembrane domains (TMDs) and two nucleotide-binding domains (NBDs). Its principal function is to facilitate transmembrane transport in the liver. Loss of ABCC6 function is a key factor in PXE pathogenesis, as demonstrated by abnormal tissue mineralization observed in ABCC6-knockdown models in vitro [17]. The proteins translated by the pathogenic mutant ABCC6 gene usually retain (or partially retain) their transporter activity, but most of them have impaired positioning and can’t reach the plasma membrane. As a result, the normal physiological functions are affected. It has been found that the reduced transport activity of ABCC6 protein leads to the reduction of intimate anti-mineralizing substances such as fetuin-A, matrix gla-protein (MGP), and serum PPi [18], ultimately resulting in connective tissue mineralization.

To date, over 2,000 unique DNA sequence variants of the ABCC6 gene have been recorded [19]. In our study, two mutation sites were identified in a Chinese child with PXE and his family: c.3412C>T and c.3160_3161del. The c.3412C>T variant is a well-documented mutation in PXE patients [14], with a reported carrier frequency of approximately 0.6% in certain populations [20]. It is located in exon 24 of ABCC6 and results in a missense substitution at amino acid position 1138, replacing arginine with tryptophan (p. Arg1138Trp). However, the c.3160_3161del mutation is a novel variant that has not been previously reported. This deletion occurs in the key structural domain exon 23 and causes a frameshift starting at threonine 1054, leading to a premature termination codon two amino acids downstream (p. Thr1054GlyfsTer2). According to the ACMG Variant Classification Guidelines [21], this variant is classified as pathogenic based on the following criteria: PVS1 (Very Strong), PM2, and PM3.

Frameshift mutations typically alter the amino acid sequence downstream of the mutation site and often introduce a premature termination codon, resulting in a truncated and dysfunctional protein [22]. If a frameshift mutation introduces a premature termination codon, the aberrant mRNA is likely to be recognized and degraded by the nonsense-mediated mRNA decay (NMD) pathway. This prevents the accumulation of the mutant transcript and subsequent translation into protein [23]. If the mutation escapes NMD, a truncated ABCC6 protein may be produced. Truncation of a critical functional domain, particularly an NBD, can directly impair the core function of the protein [24]. Given that c.3412C>T is a known pathogenic missense mutation, we propose that the compound effect of the two mutations results in a loss of functional ABCC6 protein. The transmission pattern of the detected two ABCC6 mutations is consistent with autosomal recessive inheritance.

According to the latest diagnostic consensus, if two pathogenic variants are detected, segregation analysis in the patient’s parents, siblings, and adult offspring is strongly advised to confirm t whether these variants reside on two different alleles. Molecular identification of ABCC6 pathogenic variants enables carrier testing within high-risk families and facilitates prenatal genetic testing for high-risk pregnancies, as well as preimplantation genetic testing (PGT) [14]. It is reported that carriers of a pathogenic ABCC6 variant face a markedly elevated risk for cerebrovascular events and for developing distinct PXE-associated signs, including comet-tail retinal lesions, calcifications in abdominal organs and testes, and rapidly progressing arterial disease in the lower limbs [5]. In young patients such as our proband, who may present with an early or incomplete form of PXE, long-term clinical surveillance and regular follow-up are essential to monitor disease progression and detect future systemic manifestations. Early genetic confirmation provides a critical foundation for timely intervention and personalized management.

## Conclusions

We identified a previously unreported mutation, c.3160_3161del, in the ABCC6 gene in a Chinese patient with PXE and his family members. Detection of ABCC6 gene mutations provides a foundation for elucidating the pathogenesis of PXE. Our findings support the autosomal recessive inheritance pattern of PXE and offer valuable information for genetic and prenatal counseling in affected families.
